# Mechanisms of 10-hydroxyoctadecanoic acid resistance in *Streptococcus pneumoniae*

**DOI:** 10.1128/jb.00223-25

**Published:** 2025-09-19

**Authors:** Cydney N. Johnson, Matthew W. Frank, Chrispin Chaguza, Brendan T. Morrow, Qidong Jia, Christopher D. Radka, Jason W. Rosch

**Affiliations:** 1 Department of Host-Microbe Interactions, St Jude Children's Research Hospital 5417, Memphis, Tennessee, USA; 2 Graduate School of Biomedical Sciences, St Jude Children's Research Hospital 5417, Memphis, Tennessee, USA; 3 Department of Microbiology, Immunology, and Molecular Genetics, University of Kentucky 4530, Lexington, Kentucky, USA; University of Southern California, Los Angeles, USA

**Keywords:** hydroxy fatty acid, *Streptococcus pneumoniae*, glycosyltransferase, recombinase, membrane composition, cell charge, fatty acid resistance, phase variation, lipid profile

## Abstract

Profiles of human nasal colonization consistently demonstrate that *Staphylococcus aureus* and *Streptococcus pneumoniae* can co-exist in the nasopharynx. Several studies have demonstrated the antagonist relationship between the two organisms via several molecular mechanisms, including competition for nutrients as well as via direct killing by hydrogen peroxide. During nasal colonization, the pneumococcus is in direct contact with the fatty acid *h*18:0, which is released into the extracellular environment by *S. aureus*. We report that *h*18:0 is specifically toxic to the pneumococcus among the pathogenic streptococci, providing a unique mechanism for interspecies competition during colonization. Exposure of cells to *h*18:0 revealed that *S. pneumoniae* could rapidly adapt to and overcome the observed toxicity. Whole-genome analysis revealed the mechanism underlying this resistance being linked to a truncation of a glycosyltransferase in the capsule biosynthesis locus and a genomic inversion in the phase variation locus, leading to altered cell surface charge and membrane lipid composition. These physiological differences in the resistant isolates may aid in repelling toxic, charged fatty acids such as *h*18:0 from the cell membrane.

**IMPORTANCE:**

The pneumococcus and *S. aureus* are two of the most well-characterized residents of the human nasopharynx; yet much remains unknown regarding how the two bacteria interact. Here, we describe the potential of *S. aureus*-produced *h*18:0, whose function and biological impact are still being described, to act as an interspecies competition molecule against *S. pneumoniae*, and how the pneumococcus can adapt to overcome its toxicity.

## INTRODUCTION

Bacterial oleate hydratase (OhyA) activity was first described in 1962, with its metabolic product identified as 10(*R*)-hydroxyoctadecanoic acid (*h*18:0) two years later (1, 2). OhyA genes are found in bacteria, but not mammals, and the encoded hydratases act on mammalian unsaturated fatty acids that contain either 9*Z* or 12*Z* double bonds (3–6). These proteins are critical in detoxifying mammalian unsaturated fatty acids and promoting virulence (5, 7, 8). Specifically, the hydroxy fatty acids resulting from the OhyA reactions are not used by the bacteria but are rather released into the environment (7, 8). Recently, it has been shown that the major OhyA metabolite, *h*18:0, from *Staphylococcus aureus* stimulates a transcriptional cascade in macrophages that is driven by the activation of PPARα, leading to suppression of the innate immune response and increased expression of fatty acid oxidation genes to degrade the hydroxy fatty acid signals (9, 10).

*Streptococcus pneumoniae* (the pneumococcus) is a Gram-positive coccus that usually exists as a commensal of the human nasopharyngeal mucosa but is often characterized as a pathogen due to its ability to disseminate from the nasopharynx into the middle ear space, lower respiratory tract, blood, and brain (11). Community-acquired pneumonia is the most common manifestation of invasive pneumococcal disease in those younger than five years or older than sixty-five years, despite the availability of antibiotics and vaccines (12, 13). All strains of the pneumococcus undergo phase variation, defined as the spontaneous, reversible phenotypic variation in colony opacity, ranging from translucent to opaque (14). Opaque variants have increased capsule production and decreased teichoic acid in the cell wall (15). In mice, infection with opaque variants leads to increased morbidity, suggesting that there is a strong selection for organisms with the opaque phenotype during invasive infection (15). Colonization is often associated with the translucent phenotype (16). Phase variation in the pneumococci depends on DNA inversion events among three *hsdS* genes of a conserved type I RM system, known as the *cod* locus (17), *ivr* locus (18), or *spnIII (*19). Genetic loci similar to *spnIII* have been identified in a diverse profile of bacterial genera (20–22).

It has been known for decades that mammalian fatty acids, such as oleic acid (18:1), are toxic to streptococci via destabilization of the bacterial membrane (23, 24). To combat toxic fatty acids, bacteria encode a myriad of methods to deal with these pressures, including detoxifying the fatty acids via modification or directly incorporating them into their membranes (25). *S. pneumoniae* encodes the fatty acid kinase system (FakAB) that allows for highly selective acquisition of host fatty acids found in human serum to replace *de novo* biosynthesis to promote membrane synthesis via scavenging from the host (26). *S. pneumoniae* encodes three distinct FakB proteins (FakB1, FakB2, and FakB3) that allow for the acquisition of saturated, monounsaturated, and polyunsaturated fatty acids, respectively (26). More recently, it has been reported that the milk protein alpha-lactalbumin and its equine milk protein homologue, when in complex with 18:1, exhibited bactericidal activity against pneumococci (27, 28). However, unlike many other Gram-positive bacterial pathogens, as well as closely related *Streptococcus* species (5, 29), the pneumococcus does not encode an *ohyA* homologue to prevent the accumulation of the unsaturated, antimicrobial fatty acid 18:1.

The human nasopharynx is a diverse and complex niche. Upon approval and widespread use of the pneumococcal conjugate vaccine (PCV), the nasopharyngeal microbiome has undergone disruptions in the population structure of *S. pneumoniae* (30, 31). The change in colonizing pneumococci has likely altered this niche, which may also affect *S. aureus*, another nasopharyngeal resident microbe (32–34). Models of cocolonization with both species show that both species can co-exist in the nasal passages (35), with stable dual-species biofilms able to be formed in the murine nasal passage (36). Epidemiological studies report a recent increase in cocolonization rate of the human nasopharynx, with both species detected in over 10% of patients (37, 38). However, the relationship between the two organisms seems antagonistic, and the molecular mechanisms by which they interact and compete for resources in this niche remain an active area of investigation. Studies have documented how the pneumococcus produces hydrogen peroxide to kill *S. aureus* (39) and *S. aureus* produces catalase, which can be used to combat bacterial and host-generated oxygen radicals (40). In the female genital tract, OhyA produced by some lactobacilli sequesters 18:1 for phospholipid biosynthesis as *h*18:0 through an OhyA-dependent mechanism, enhancing bacterial fitness. Only organisms that encode OhyA can utilize *h*18:0, while *Lactobacillus* species lacking OhyA are competitively disadvantaged (41). As a resident of the nasal microbiota, the pneumococcus is in direct contact and competition with bacteria such as *S. aureus* that encode an OhyA and release *h*18:0 into the environment (7, 42). We postulated that *h*18:0 released by *S. aureus* could be a potential mechanism by which this major human pathogen could eradicate competing bacterial species, specifically those lacking a homolog of OhyA such as *S. pneumoniae*.

Here, we report that *S. aureus*-derived *h*18:0 is toxic to the *S. pneumoniae*, a feature not widely shared by related pathogenic streptococci. Despite strong inhibitory activity, under selective pressure, *S. pneumoniae* rapidly developed resistance to *h*18:0. Genetic analysis of resistant mutants uncovered the genetic basis for *h*18:0 fatty acid resistance which involved the mutation of a glycosyltransferase and a genomic rearrangement in the phase variation locus *spnIII* that is likely driven by a recombinase. The genetic changes in the resistant mutants altered both the surface charge of the resistant strains and modulated the lipid composition of their cellular membranes. These data underscore the potential role of *h*18:0 in mediating microbial competition during colonization and bacterial strategies deployed to circumvent toxicity of this molecule.

## RESULTS

### 10-Hydroxyoctadecanoic acid is toxic to the pneumococcus

**Fig 1:**
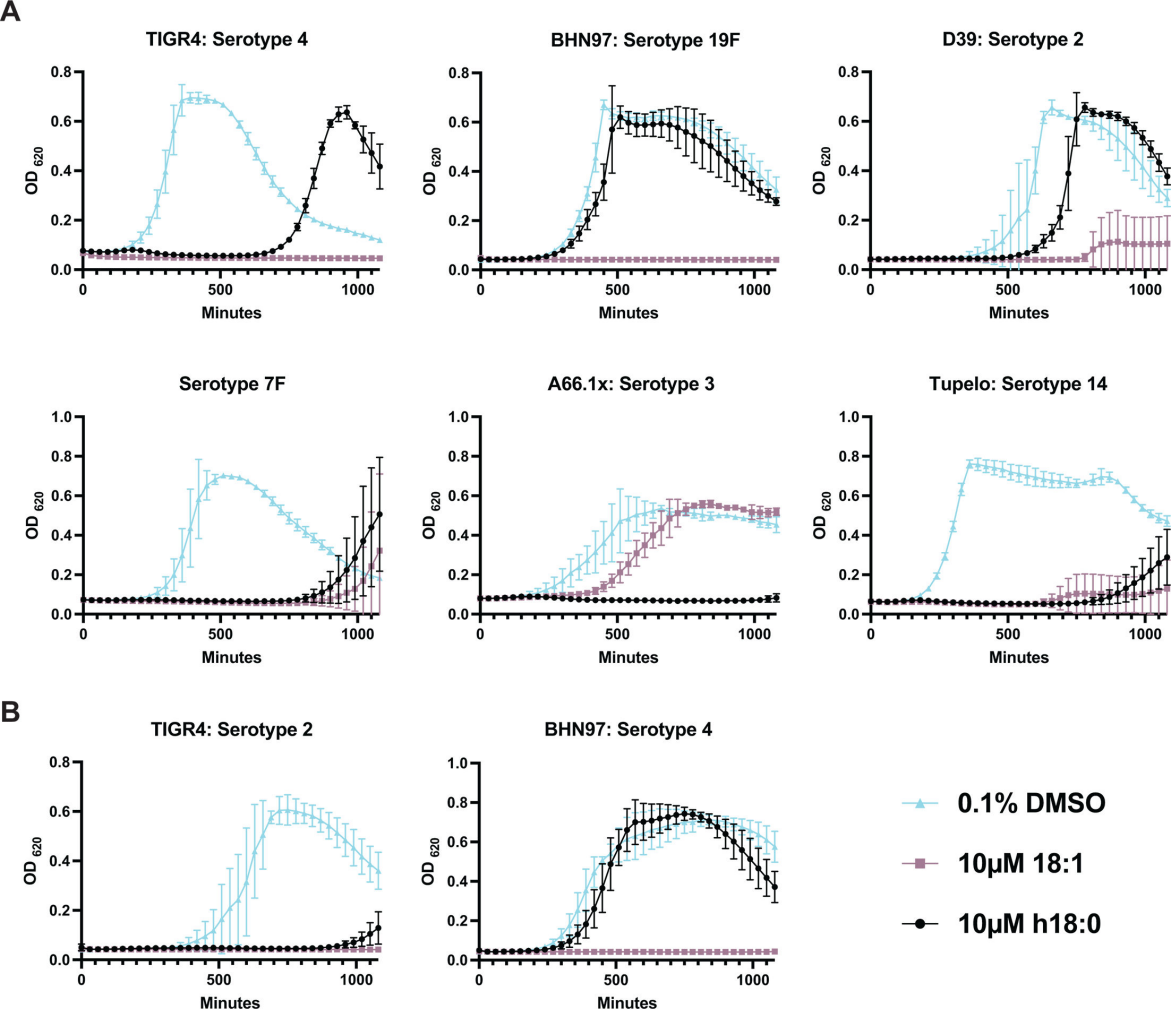
*h*18:0 exhibits bactericidal activity against pneumococcal capsule types associated with invasive disease. (**A**) Pneumococci were grown in complex media with 0.1% DMSO, 0.1% DMSO + 10 µM 18:1, or 0.1% DMSO + 10 µM h18:0. Titles above the graph designate strain name: serotype. For serotype 7F, there is no strain name. Growth was evaluated by measuring optical density every 30 minutes for 20 hours. Serotypes are denoted above each graph. All experiments were done in biological triplicate with technical triplicates, except 7F and A661.x, which were performed in biological duplicate with technical triplicates. (**B**) Capsule switch mutants of TIGR4 and BHN97 grown in 0.1% DMSO, 0.1% DMSO + 10 µM 18:1, or 0.1% DMSO + 10 µM h18:0. Mean and standard deviation values are shown.

Oleic acid, the fatty acid 18:1, causes toxicity to several streptococcal species via membrane disruption (23, 24), although the extent to which this extends to the pneumococcus remains unknown. *S. aureus* converts 18:1 to *h*18:0 via OhyA; however, *S. pneumoniae* lacks this protein. We sought to determine whether the host-derived precursor and the bacterially modified fatty acids produced by the *S. aureus* OhyA reaction are toxic to pneumococci. *S. pneumoniae* is composed of over 100 different serotypes that are determined by the polysaccharide capsule produced by the bacteria. Pneumococcal strains TIGR4 (serotype 4), BHN97 (serotype 19F), D39 (serotype 2), Serotype 7F, A66.1x (serotype 3), and Tupelo (serotype 14) were grown in C + Y media supplemented with solute alone (DMSO) and either 10 µM 18:1 or 10 µM h18:0. *S*. aureus, when grown in 100% human serum, produces on average 27 µM h18:0^7^. Additionally, 10 µM h18:0 has been used in other studies to explore the impact of *h*18:0 on immune cell biology (9). Therefore, we chose to utilize 10 µ*M* h18:0 in our experiments. There was considerable heterogeneity in how different strain backgrounds responded to the supplemented fatty acid (Fig. 1A). All strains tested were susceptible to 18:1 toxicity except A661.x (serotype 3), which was inherently resistant, confirming the broadly inhibitory nature of 18:1 observed in several bacterial species (23–25, 41).

The susceptibility of strains to *h*18:0 susceptibility was more heterogeneous across strains and capsule backgrounds. D39, like TIGR4, was susceptible to *h*18:0 toxicity but established normal growth after a prolonged lag phase. Strains 7F, A66.1x, and Tupelo largely remained susceptible to *h*18:0 toxicity. The pneumococcal capsule is a highly diverse structure in terms of both composition and charge, both of which play important roles in bacterial fitness under various conditions. To determine whether the phenotypes rely upon the differences between capsule types, we generated capsule-switched variants of TIGR4 expressing the 19F capsule and BHN97 expressing the type 4 capsule. The capsule-switched strains maintained the susceptibility profile of the parental strains expressing their native capsule (Fig. 1B), indicating that differing susceptibility to 18:1 and *h*18:0 was not related to the different polysaccharide capsules being produced but rather another genetic determinant. There is a difference in the growth of the TIGR4 capsule switch compared to WT TIGR4, which we hypothesize is due to the new metabolic burden of producing a nonsynonymous capsule.

### 10-Hydroxyoctadecanoic acid toxicity is specific to closely related *Streptococcus* species

**Fig 2:**
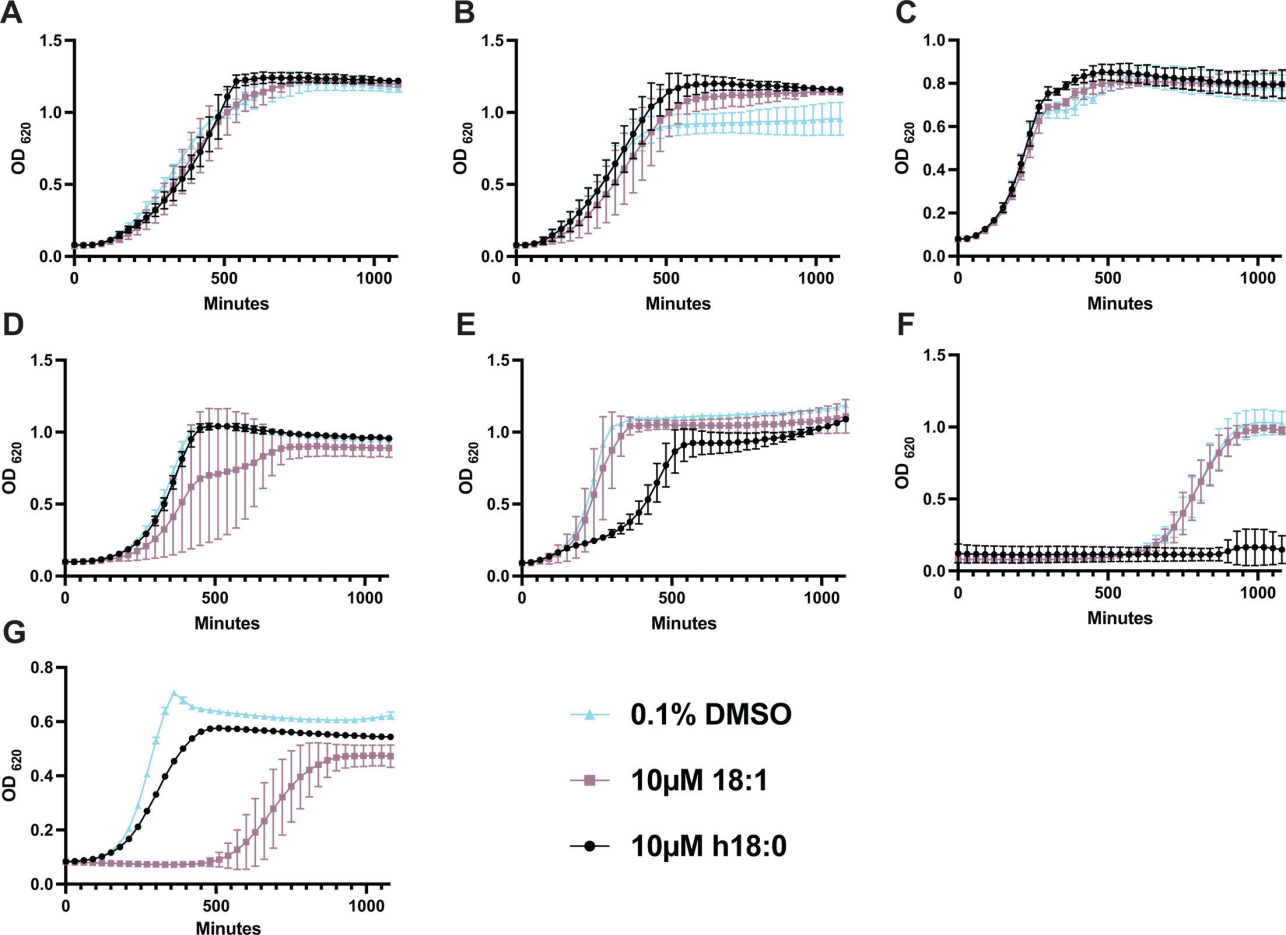
h18:0 toxicity is not conserved across Gram-positive species. Bacterial strains were grown in media supplemented with 0.1% DMSO, 0.1% DMSO + 10 µM 18:1, or 0.1% DMSO + 10 µM h18:0. All experiments were done in biological triplicate with technical triplicates. (**A**) *S. aureus*, (**B**) *S. aureus* D*ohyA*, (**C**) *E. faecalis,* (**D**) *S. mutans*, (**E**) *S. agalactiae,* (**F**) *S. pyogenes,* and (**G**) *S. mitis*. Mean and standard deviation values are shown.

*S. pneumoniae* does not encode a gene for producing OhyA, but other closely related Gram-positive bacteria do. According to published genomes, *S. pyogenes* (KEGG T00050, Spy_0470) and *Enterococcus faecalis* (KEGG T00123, EF3303), once classified as group D streptococci; *Streptococcus mutans* (KEGG T00100, SMU_515 and SMU_1584 c); and *Streptococcus agalactiae* (KEGG T00091, SAG1508) all encode for a predicted functional oleate hydratase. According to the published genome for *Streptococcus mitis* B6 (KEGG T01173), *S. mitis* does not encode an OhyA homologue, such as the pneumococcus. *S. pyogenes* is known to be susceptible to 18:1 toxicity (24), but the susceptibility of the other species has not been determined. Therefore, we next asked whether these closely related bacteria are susceptible to 18:1 and *h*18:0 toxicity using representative strains (Fig. 2). As expected, *S. aureus* was resistant to 18:1 and *h*18:0 toxicity in an OhyA-independent manner (Fig. 2A and B). *E. faecalis, S. mutans*, and *S. agalactiae* were not killed by either 18:1 or *h*18:0 (Fig. 2C through F). *S. pyogenes* was susceptible only to *h*18:0 (Fig. 2F). Surprisingly, *S. mitis* did not exhibit toxicity to *h*18:0; however, it was the only tested species that did display some toxicity when grown in 18:1. These data underscore that even among closely related streptococcal species, sensitivity of potential antimicrobial fatty acids varies considerably.

### Sequencing reveals that resistance to 10-hydroxyoctadecanoic acid relies on FabT

**Fig 3:**
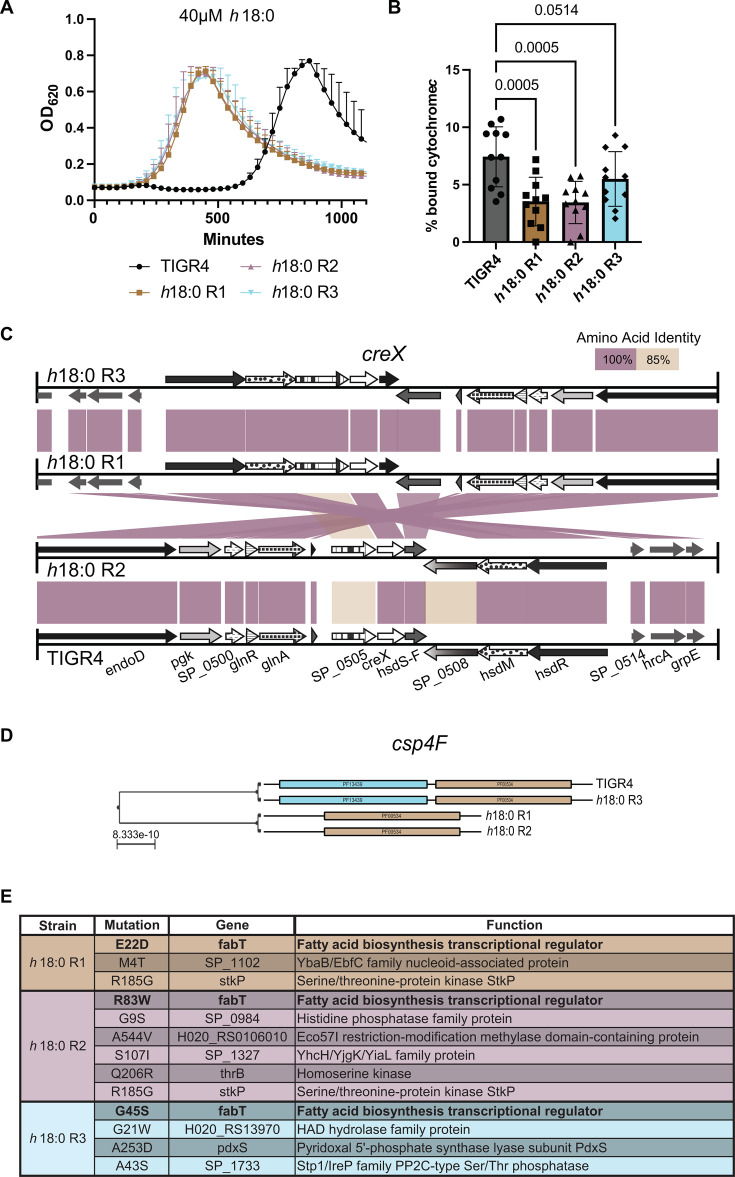
Mutation of a glycosyltransferase or genetic recombination can cause *h*18:0 resistance. (**A**) *h*18:0-resistant mutants can grow in 40 µM h18:0. Wild-type TIGR4 is susceptible to the toxicity. Mean and standard deviation values are shown. (**B**) Resistant mutants exhibit a more positive cell charge due to decreased binding of cytochrome *c*, a positively charged protein. Mean and standard deviation values are shown. *P* values are reported on the graph. Statistical significance was determined by one-way ordinary ANOVA. (**C**) Genomic analysis of the locus around *creX* reveals an inversion (crossed amino acid identity lines between genomes) and mutation of genes flanking *creX*. Homologous genes display identical patterns. Lines between genes represent amino acid similarity. (**D**) *cpsF4* domain visualization among the four genomes. (**E**) SNPs identified in the *h*18:0 resistant mutants that result in amino acid changes.

Our growth curve data in Fig. 1 suggest that prolonged exposure to *h*18:0 selects for resistant mutants within the pneumococcal population. Specifically, we isolated three TIGR4 clones resistant to *h*18:0 from three independent experiments. These clones demonstrated normal growth kinetics in standard C + Y media that was the base media for all experiments (data not shown). Supplementation of media with 40 µM h18:0 confirmed that all three clones demonstrated increased resistance to the inhibitory activity of *h*18:0 (Fig. 3A), indicating a heritable resistance mechanism. We next sought to determine if the resistant clones demonstrated physiological characteristics that could explain the observed resistance patterns against *h*18:0. Hydroxy fatty acids are typically negatively charged due to the carboxylic acid group. While our capsule-switched mutants did not demonstrate appreciable difference in *h*18:0 sensitivity, we postulated that capsule-independent modulation of surface charge could be involved in resistance. This was measured using a cytochrome *c* binding assay, which is widely utilized to measure relative surface charge (43–45). We found that these clones exhibited a more positively charged cell surface, as determined by cytochrome *c* binding, compared with the sensitive parental strain (Fig. 3B).

To determine the genetic basis of this resistance, the *h*18:0-resistant mutants (strains R1, R2, and R3) were subjected to both short-read Illumina and long-read PacBio sequencing. Genomes were assembled *de novo* and analyzed using zDB (46) (Fig. 3C). We observed mutations surrounding the site-specific recombinase-encoding gene *creX* (SP_0506) in the *h*18:0-resistant mutants (Fig. 3C). The gene *creX*, along with the restriction modification system *hsdM* and *hsdR*, is part of the *spnIII* locus, which is highly conserved across pneumococcal strains and is involved in phase variation (19). Specifically, *h*18:0-resistant clones R1 and R3 have undergone inversions of this locus that centers around *creX*. Unlike the other resistant isolates, *h*18:0-resistant clone R2 had mutations in the genes SP_0505 and SP_0508, which are upstream and downstream of *creX*, respectively. These genes encode proteins that are about 85% identical at an amino acid level to the TIGR4 protein, which was also sequenced at the time we performed these experiments to provide an up-to-date reference genome for our analysis. SP_0505 and SP_0508 (*hsdS*) are putative subunit S proteins of type I restriction–modification systems. CreX-driven recombination of *hsdS* is a known rearrangement that leads to phase variation in pneumococci (47). We attempted to quantify the ratio of opaque to transparent colonies in our *h*18:0 resistant clones following previously published methods; however, we were not successful.

We also queried for additional genetic features unique to the *h*18:0-resistant clones. *csp4F* (SP_0351), a glycosyltransferase in the capsule biosynthesis locus (Fig. 3D), was truncated in *h*18:0-resistant clones R1 and R2, having deleted the 5′ end of this gene. Despite these mutations, these strains still produced type 4 capsule as determined by latex agglutination and ELISA (data not shown). SNP analysis revealed diverse mutations across the three resistant strains; however, all three mutants have been selected for mutations in *fabT* (Fig. 3E). R1 has a mutation at E22D, R2 at R83W, and R3 at G45S. FabT is a MarR family transcriptional repressor of the *de novo* fatty acid biosynthesis locus (48). The E22D mutation in R2 lies in the first alpha helix domain of the protein. G45S is in the second alpha helix of the N-terminal helix-turn-helix motif (49). Helix-turn-helix domains are generally involved in DNA binding. R83W is at the end of the first beta sheet. Beta sheets in DNA-binding proteins can interact with the major groove of the target DNA, increasing contact between the transcriptional regulator and the target DNA (50). Our results suggest that resistance to *h*18:0 can likely arise via mutation of *fabT*, although future mechanistic work is necessary to confirm these preliminary findings.

### *S. pneumoniae* that are resistant to 10-hydroxyoctadecanoic acid have altered membrane composition

**Fig 4:**
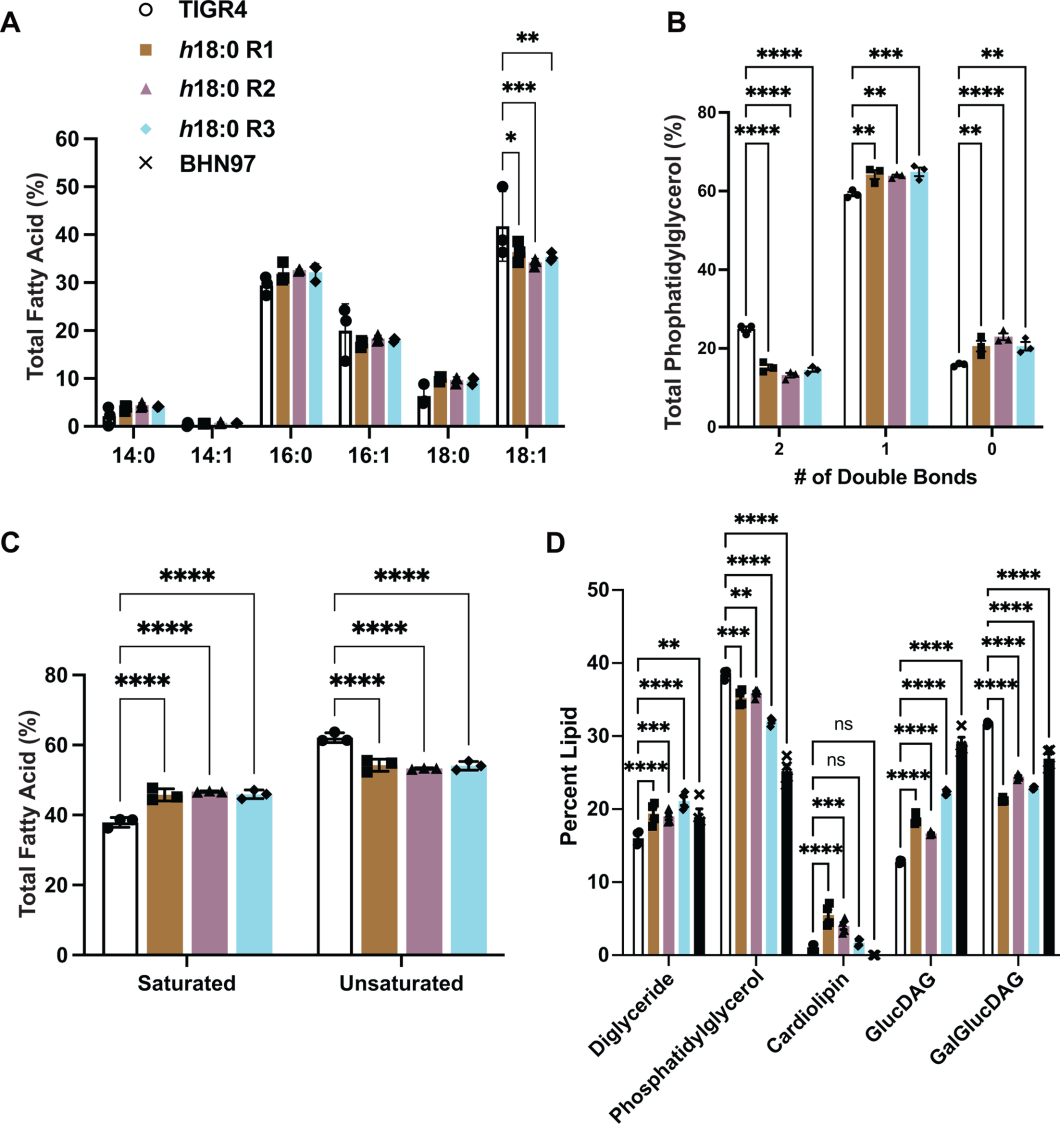
Pneumococci resistant to h18:0 have altered membrane lipid composition. (**A**) Fatty acid composition of the membrane lipids composition was determined for wild-type and *h*18:0-resistant TIGR4 strains. (**B**) PG species are grouped by the number of double bonds they contain in the fatty acids. (**C**) From the FAME analysis, fatty acids are grouped together as saturated or unsaturated fatty acids. (**D**) Acetate labeling was performed to determine the lipid composition of the cell membranes. Mean and standard deviation values from biological triplicates are shown for all panels. Statistical significance was determined by one-way ordinary ANOVA. *P ≤ 0.05, **P ≤ 0.01, ***P ≤ 0.001, ****P ≤ 0.0001, ns = not significant.

Previous studies have implicated changes in lipid composition when bacteria are grown with fatty acids (51–53). Phase variation affects lipid and cell wall composition (54, 55). Therefore, we determined whether our *h*18:0-resistant clones with genomic rearrangements in the *spnIII* locus, which is involved in phase variation, have altered lipid profiles in their cell membranes. Utilizing gas chromatography, we quantified the fatty acid composition of the lipid membrane in wild-type TIGR and the h18:0-resistant clones (Fig. 4A). The *h*18:0-resistant clones had significantly less 18:1 and more 18:0 in their lipids compared to WT. The h18:0-resistant clones contained significantly more saturated and fewer unsaturated fatty acids in their membrane than WT clones did (Fig. 4B). Additionally, the phosphatidylglycerol (PG) profiles for wild-type and resistant strains were analyzed by mass spectrometry. The different PG species contain a total of zero, one, or two double bonds in their fatty acid chains. In the total PG, the h18:0-resistant strains contained a lower percentage of PG species that contained two double bonds, such as 36:2, and an increased percentage with zero or one double bond (Fig. 4C). Using [1-^14^C]acetate labeling, we characterized the lipid profile of TIGR4, the *h*18:0-resistant clones, and BHN97 (Fig. 4D). BHN97 was included in this assay to determine whether the lipid profiles of the *h*18:0-resistant clones looked similar to BHN97, a strain of the pneumococcus that we identified as inherently resistant to *h*18:0 (Fig. 1). The resistant clones have significantly increased amounts of diacylglycerol and glucosyl-diacylglycerol, while phosphatidylglycerol and galactosyl-glucosyl-diacylglycerol amounts are decreased, which is similar to BHN97. In conclusion, *h*18:0-resistant clones have different lipid compositions, both in the fatty acid composition of their lipids and the amount of different lipid species, compared to wild-type *S. pneumoniae*.

## DISCUSSION

The human nasopharynx is a diverse and complex niche that promotes constant interactions between bacteria to compete for limited space and nutrients. The pneumococcus and *S. aureus* are two of the most well-known residents of the human nasopharynx; yet much remains unknown regarding how the two bacteria interact. The *S. aureus* oleate hydratase OhyA converts host-derived 18:1 fatty acid into *h*18:0^8^. *h*18:0 is released from the bacterial cells as a free fatty acid and would, therefore, be presented to the pneumococcus as an unesterified fatty acid. Yet, no evidence indicates that host cells can integrate *h*18:0 into a lipid, raising the question as to what the impact of this fatty acid is on the neighboring microbiota. In this study, we discovered that *h*18:0 is toxic to the pneumococcus, supporting the idea that *h*18:0 may serve as a secreted metabolite used by *S. aureus* to mediate competition among other bacterial species. We decided to determine the breadth of *h18*:0 toxicity across other streptococcal species. This fatty acid does not exhibit toxicity toward *Enterococcus faecalis* (formerly Group D streptococci), *Streptococcus mutans* (Viridans group streptococci), and *Streptococcus mitis* (Viridans group streptococci). *Streptococcus agalactiae* (Group B streptococci) exhibits a shift in the growth curve, suggesting some toxicity from *h*18:0. *Streptococcus pyogenes* (Group A streptococci) was the only other tested species that was susceptible to *h*18:0 toxicity, as evidenced by the absence of obvious growth.

While we observed that *h*18:0 effectively inhibited growth of most pneumococcal strains, we noted that resistance consistently and readily emerged against this antimicrobial fatty acid. While most pneumococcal genetic backgrounds and serotypes were sensitive to *h*18:0, there were notable exceptions. For example, A66.1x, a serotype 3 strain, was the only tested pneumococcal serotype with complete resistance to 18:1. Serotype 3 infections are characterized as having severe clinical manifestations and causing invasive disease, including empyema, bacteremia, cardiotoxicity, and meningitis, with a fatality rate over 30% (56). Resistance to 18:1 by serotype 3 may be due to the highly mucoid capsule that this serotype produces. After generating TIGR4 clones resistant to *h*18:0, subsequent analysis of the lipid profile of the *h*18:0-resistant mutants revealed subtle but consistent alterations in lipid membrane composition. The *h*18:0-resistant clones contained significantly more saturated and fewer unsaturated fatty acids in their membrane than the parental wild-type strains. In addition, the resistant clones have significantly increased amounts of diacylglycerol and glucosyl-diacylglycerol, while phosphatidylglycerol and galactosyl-glucosyl-diacylglycerol amounts are decreased, such as BHN97. This is in agreement with the measurement of surface charge differences in the resistant mutants, suggesting that electrostatic repulsion may confer a protective benefit against the toxicity of *h*18:0. There is also the noteworthy observation that *S. pneumoniae* does not incorporate *h*18:0 into their membranes, meaning either their FakB proteins do not accept *h*18:0 or their lipid biosynthetic machinery does not accept *h*18:0 acyl chains. Future studies are necessary to clarify the mechanistic relationship between membrane lipid composition and fatty acid resistance.

The genetic basis of fatty acid resistance has been difficult to determine over the last few decades. Many of the studies have utilized sequencing to examine a selected subset of genes, generally the Fak locus, because inactivation of genes in this pathway can lead to fatty acid resistance (57), and have found single mutations that lead to fatty acid resistance (58). In this study, we used whole-genome sequencing for our resistant clones to take an unbiased approach to discovering genetic mechanisms underlying *h*18:0 resistance. By using both short- and long-read sequencing, we were able to generate *de novo* genomes of our *h*18:0-resistant clones with sufficient coverage and depth. This led to the elucidation that in *S. pneumoniae* TIGR4, *h*18:0 resistance can come from multiple mechanisms. Two of the three clones had large genomic inversions surrounding *creX. creX* is part of the phase variation–determining locus *spnIII*, which is highly conserved across pneumococcal strains; genomic recombination of this locus alters locus expression and controls phase variation (19). The zDB analysis only enabled examination of the 20 kB surrounding this gene, so the inversion may be larger than observed. Intriguingly, it has been reported that some serotype 19F strains lack *creXI* (59)*,* which may explain why BHN97, which also lacks a *creX*, is resistant to *h*18:0 independent of the expressed capsule type. Unfortunately, despite the availability of published protocols, we were unable to determine via microscopy if these resistant strains were phase-locked (60).

Truncation of *cps4F* in *h*18:0-resistant clones R1 and R2 would presumably lead to an inactive glycosyltransferase. In a study surveying pneumococcal isolates from Malaysia, *cps4F* was conserved across the sequenced serotype 4 isolates (61). Despite these mutations, the strains expressed serotype 4 capsule as determined by latex agglutination assays. Differences in glycosylation patterns have the potential to change cell surface potential, which may aid in repelling charged fatty acids from the cell membrane. Increasing cell charge inhibits the ability of fatty acids to permeate the cell wall, which will inhibit access to the sites of action on the inner membrane (42, 62, 63).

FabT is a transcriptional repressor of the fatty acid synthesis pathway conserved across streptococcal species. It is comprised of six alpha helices and three beta sheets and exists as a homodimer. In our mutants, we observed three different mutations: E22D in R1, G45S in R3, and R83W in R2. The 22nd and 45th amino acids lay in the first two alpha helices, respectively (49). Helix α1 of one subunit and helices α5′ and α6′ of the other subunit intertwine, forming FabT, a homodimer. Helices α2, α3, and α4 of the winged helix-turn-helix domain are responsible for the DNA-binding activity. A second wing motif is made up of an antiparallel β-hairpin (β1–β2), in which the 83rd amino acid is found at the end of the first beta sheet. The two winged helix-turn-helix domains are located at the base of FabT’s pyramidal architecture and clamp the target DNA on both sides (49). It is highly possible that the three mutations we observed in this study impact the ability of FabT to bind to its target sequence. The main caveat of this study is that we have not generated the mutations we identified in the native TIGR4 background to demonstrate that they are solely responsible for *h*18:0 resistance. Despite having different identified genetic mutations, all the *h*18:0-resistant mutants had altered composition of lipid species in their cell membranes. Because of the intrinsic resistance to *h*18:0, future studies exploring the mechanism of resistance in BHN97 may uncover additional mechanisms of fatty acid resistance.

## MATERIALS AND METHODS

### Bacterial strains and growth conditions

Pneumococcal strains were grown on tryptic soy agar (Sigma Aldrich) supplemented with 3% defibrinated sheep blood and 20 µg/mL neomycin. *S. aureus* AH1263 (64), AH1263 Δ*ohyA* (8), *E. faecalis* OG1RF, *Streptococcus mutans* UA159*, Streptococcus agalactiae* 2603, and *Streptococcus pyogenes* HSC5 were grown in Todd-Hewitt media (BD) supplemented with 2% (wt/vol) yeast extract (Gibco). Pneumococcal cultures were inoculated from newly streaked TSA blood agar plates into C + Y, a semi-synthetic casein liquid media with 0.5% (wt/vol) yeast extract (65), and grown at 37°C in 5% CO_2_. Growth curve measurements were read in a 96-well plate using a Biotek Synergy, with starting OD_620_ of 0.05–0.1 for all strains.

### Cytochrome *c* binding assay

The cytochrome *c* binding assay was modeled after previously published assays (45, 66). Pneumococci were grown to an OD_620_ of 0.4 in 7 mL C + Y. One milliliter of the culture was pelleted at 13,000 rpm for 1 minute and resuspended in 400 µL of 1 M sterile 4-(2-hydroxyethyl)−1-piperazineethanesulfonic acid (HEPES, Sigma Aldrich). Cytochrome *c* (Sigma) was added to a final concentration of 0.5 mg/mL. The cell mixture was incubated at room temperature for 10 minutes, followed by centrifuging the bacteria at 13,000 rpm for 1 minute. The assay was performed in biological triplicate, and the experiment was repeated three times. The amount of cytochrome *c* remaining in the supernatant was quantified by measuring absorbance at 535 nm and compared to samples with no bacteria.

### DNA extraction, sequencing, and genomic assembly

DNA was extracted via phenol/chloroform extraction from wild-type *S. pneumoniae* TIGR4, and from three independently derived *h*18:0-resistant mutants, which were grown to stationary phase in either 0.01% DMSO (wild-type) or 0.01% DMSO +10 µM h18:0 (resistant mutants). High-molecular-weight DNA was submitted for Illumina short-read sequencing on the HiSeq platform and PacBio Revio long-read sequencing performed by the Hartwell Center at St. Jude Children’s Research Hospital. For genome assembly, 50,000 PacBio reads were randomly selected from each sample using seqtk, and Hifiasm was used to assemble the genomes (67). The resulting assemblies were then annotated using Prokka (68). We used two approaches to assess potential genomic differences distinguishing the parental *S. pneumoniae* strain from those resistant to *h*18:0. First, we identified and compared the presence and absence of the single-nucleotide polymorphisms (SNPs) between the parental and resistant strains. To do this, we generated a consensus whole-genome alignment of the parental and three resistant strains by mapping their sequence reads against the TIGR4 reference genome (GenBank accession: NZ_AKVY01000001) using Snippy (69) (version 4.6.0) and then identified positions containing SNPs using Snp-Sites (version 2.5.1) (70). We then annotated the identified SNPs to determine the associated genomic features (genes, tRNA, and intergenic regions), their gene products, and the effect of the mutations on the encoded amino acids. As a complementary approach, we compared the distribution of variable-length sequence substrings (unitigs) generated from the assembled genomes of the four strains using PacBio long-read sequencing data, which were assembled using hifiasm (version 0.23.0-r691) (67). We identified and queried the presence and absence of the unitigs using Bifrost (version 1.0.1) (71). We considered genetic changes (SNPs or unitigs) associated with resistance to *h*18:0 as those present in all the resistant strains and absent in the parental strain. Sequences have been uploaded to NCBI under the BioProject ID PRJNA128543.

### Mass spectrometry of phosphatidylglycerol

Wild-type and *h*18:0-resistant TIGR4 strains were grown in C + Y media for 6 hours. After which, cells were pelleted, and the supernatant was removed. Cell pellets were stored at −20°C until analysis. Lipids were extracted from the cells by using the Bligh and Dyer method (72). Lipid extracts were resuspended in chloroform:methanol (1:1). PtdGro was analyzed by using a Shimadzu Prominence UFLC attached to a QTrap 4500 equipped with a Turbo V ion source (Sciex). Samples were injected onto an Acquity UPLC BEH HILIC (1.7 µm, 2.1 × 150 mm column; Waters) at 45°C with a flow rate of 0.2 mL/min. Solvent A was acetonitrile, and solvent B was 15 mM ammonium formate (pH 3). The HPLC program was the following: starting solvent mixture of 96% A/4% B, 0 to 2 minutes isocratic with 4% B; 2 to 20 minutes linear gradient to 80% B; 20 to 23 minutes isocratic with 80% B; 23 to 25 minutes linear gradient to 4% B; and 25 to 30 minutes isocratic with 4% B. The QTrap 4500 was operated in the Q1 negative mode. The ion source parameters for Q1 were as follows: ion spray voltage, −4500V; curtain gas, 25 psi; temperature, 350°C; ion source gas 1, 40 psi; ion source gas 2, 60 psi; and declustering potential, −40V. The system was controlled, and the data were analyzed by the Analyst software (Sciex). The sum of the areas under each peak in the mass spectra was calculated, and the percentage of each molecular species present was calculated with LipidView software (Sciex).

### Fatty acid analysis by gas chromatography

Fatty acid methyl esters were prepared from the lipid extracts using anhydrous methanol/acetyl chloride. The fatty acid methyl esters were analyzed using a Hewlett-Packard model 5890 gas chromatograph equipped with a flame ionization detector and separated on a 30 m × 0.536 mm × 0.50 μm DB-225 capillary column (Agilent). The injector was set at 250°C, and the detector was set at 300°C. The temperature program was as follows: an initial temperature of 70°C for 2 minutes, a ramp rate of 20 °C/min for 5 minutes (final temperature, 170°C), a ramp rate of 2 °C/min for 10 minutes (final temperature, 190°C), a hold at 190°C for 5 minutes, a ramp rate of 2 °C/min for 15 minutes (final temperature, 220°C), and a hold at 220°C for 5 minutes. The identity of each fatty acid methyl ester was determined by comparing its retention time with fatty acid methyl ester standards (Matreya). The composition was expressed as weight percentages.

### [1-^14^C] Acetate Incorporation into Lipids

When strains grown in C + Y media under standard growth conditions reached an OD_620_ of 0.2, 20 µCi of [1-^14^C] acetate was added and incubated at 37°C to an OD_620_ of 0.8. Cells were collected by centrifugation and washed twice with PBS and once with water. Lipids were extracted using the Bligh and Dyer method. Radiolabeled lipids were quantified by scintillation counting. The lipids were separated by loading equivalent amounts of radioactivity onto a silica gel H thin-layer plate and were developed in ethanol:chloroform:triethylamine:water:0.5M EDTA (34:30:35:6.5:0.210, vol/ vol/vol/vol/vol). The radiolabeled lipids were visualized using the Typhoon FLA 9500 (GE Healthcare). Lipids were identified with known standards.

### Statistical analyses

All statistical analyses were performed in GraphPad Prism version 10.3.1. In each figure legend, we include the statistical method that was performed.
